# Advancing neuropsychiatric genetics training and collaboration in Africa

**DOI:** 10.1016/S2214-109X(18)30042-1

**Published:** 2018-03-01

**Authors:** Celia van der Merwe, Emmanuel K Mwesiga, Nathaniel W McGregor, Abebe Ejigu, Abigiya Wondimagegnehu Tilahun, Allan Kalungi, Benedict Akimana, Benyam Worku Dubale, Felicita Omari, Jackline Mmochi, Lerato Majara, Linnet Ongeri, Melkam Alemayehu, Nastassja Koen, Shareefa Dalvie, Symon M Kariuki, Michelle Hoogenhout

**Affiliations:** GINGER, Harvard T H Chan School of Public Health, Boston, MA, USA; Stanley Center for Psychiatric Research at the Broad Institute of MIT and Harvard, Cambridge, MA, USA; University of Cape Town, Cape Town 7935, South Africa; GINGER, Harvard T H Chan School of Public Health, Boston, MA, USA; Stanley Center for Psychiatric Research at the Broad Institute of MIT and Harvard, Cambridge, MA, USA; University of Cape Town, Cape Town 7935, South Africa; Makerere University, Kampala, Uganda; GINGER, Harvard T H Chan School of Public Health, Boston, MA, USA; Stanley Center for Psychiatric Research at the Broad Institute of MIT and Harvard, Cambridge, MA, USA; Stellenbosch University, South Africa; GINGER, Harvard T H Chan School of Public Health, Boston, MA, USA; Stanley Center for Psychiatric Research at the Broad Institute of MIT and Harvard, Cambridge, MA, USA; Addis Ababa University, Addis Ababa, Ethiopia; GINGER, Harvard T H Chan School of Public Health, Boston, MA, USA; Stanley Center for Psychiatric Research at the Broad Institute of MIT and Harvard, Cambridge, MA, USA; Makerere University, Kampala, Uganda; Stellenbosch University, South Africa; Mental Health Project of the Medical Research Council/Uganda Virus Research Institute, Entebe, Uganda; GINGER, Harvard T H Chan School of Public Health, Boston, MA, USA; Stanley Center for Psychiatric Research at the Broad Institute of MIT and Harvard, Cambridge, MA, USA; Makerere University, Kampala, Uganda; GINGER, Harvard T H Chan School of Public Health, Boston, MA, USA; Stanley Center for Psychiatric Research at the Broad Institute of MIT and Harvard, Cambridge, MA, USA; Addis Ababa University, Addis Ababa, Ethiopia; GINGER, Harvard T H Chan School of Public Health, Boston, MA, USA; Stanley Center for Psychiatric Research at the Broad Institute of MIT and Harvard, Cambridge, MA, USA; Moi Teaching and Referral Hospital, Eldoret, Kenya; GINGER, Harvard T H Chan School of Public Health, Boston, MA, USA; Stanley Center for Psychiatric Research at the Broad Institute of MIT and Harvard, Cambridge, MA, USA; Moi University, Eldoret, Kenya; GINGER, Harvard T H Chan School of Public Health, Boston, MA, USA; Stanley Center for Psychiatric Research at the Broad Institute of MIT and Harvard, Cambridge, MA, USA; University of Cape Town, Cape Town 7935, South Africa; GINGER, Harvard T H Chan School of Public Health, Boston, MA, USA; Stanley Center for Psychiatric Research at the Broad Institute of MIT and Harvard, Cambridge, MA, USA; Kenya Medical Research Institute, Nairobi, Kenya; GINGER, Harvard T H Chan School of Public Health, Boston, MA, USA; Stanley Center for Psychiatric Research at the Broad Institute of MIT and Harvard, Cambridge, MA, USA; Addis Ababa University, Addis Ababa, Ethiopia; GINGER, Harvard T H Chan School of Public Health, Boston, MA, USA; Stanley Center for Psychiatric Research at the Broad Institute of MIT and Harvard, Cambridge, MA, USA; University of Cape Town, Cape Town 7935, South Africa; Stellenbosch University, South Africa; GINGER, Harvard T H Chan School of Public Health, Boston, MA, USA; Stanley Center for Psychiatric Research at the Broad Institute of MIT and Harvard, Cambridge, MA, USA; University of Cape Town, Cape Town 7935, South Africa; GINGER, Harvard T H Chan School of Public Health, Boston, MA, USA; Stanley Center for Psychiatric Research at the Broad Institute of MIT and Harvard, Cambridge, MA, USA; Clinical Research (Neurosciences), KEMRI-Wellcome Trust Research Programme, Kilifi, Kenya; University of Oxford, Oxford, UK; GINGER, Harvard T H Chan School of Public Health, Boston, MA, USA; Stanley Center for Psychiatric Research at the Broad Institute of MIT and Harvard, Cambridge, MA, USA; University of Cape Town, Cape Town 7935, South Africa

Neuropsychiatric disorders are a major contributor to functional impairment and mortality in low-income and middle-income (LMIC) settings, such as sub-Saharan Africa.^1^ Given the increasing population size and life expectancy in this region, years lived with disabilities associated with psychiatric diseases are estimated to double in the next 30 years.^2^ To compound the severity of these statistics, available treatment options are largely ineffective, only yielding desirable results in about 50% of cases.^3^

Despite the clinical burden and high heritability of neuropsychiatric disorders, their genetic backgrounds are poorly understood. Furthermore, populations of African ancestry are substantially under-represented in global research of neuropsychiatric genetics.^4–6^ From a scientific perspective, the study of modern African genomes might provide key insights into gene discovery and mapping of disease-associated variants.^7,8^ The genetics of African populations, which feature increased allelic variability and reduced linkage disequilibrium compared with European populations, might reveal the missing layer of human variation that arose between 100 000 and 5 million years ago.^9^ From a clinical perspective, discovering the genetic make-up of these populations is integral to addressing global inequities in neuropsychiatric service delivery and health-care infrastructure and to translating empirical data into public health interventions in sub-Saharan Africa.

To date, advancements in neuropsychiatric genetics research in sub-Saharan Africa have been limited by low capacity—funding availability, appropriate infrastructure, and qualified researchers are under-represented in African institutions compared with counterparts in high-income countries (HICs). To improve representation of African institutions in neuropsychiatric genetics research, researchers and funding bodies should prioritise projects involving collaborations between institutions in HICs and LMICs that contain a capacity-building component. Initiatives such as Human Hereditary and Health in Africa (H3Africa), the International Brain Research Organization (IBRO), and the Wellcome Trust Developing Excellence in Leadership, Training, and Science Initiative (DELTAS) programme have been invaluable in building these collaborations and in providing genomics-related research training opportunities for researchers from African institutions.^10^ Training events, online courses, and workshops have provided unprecedented learning strategies for medical, bioinformatics, and public health education in LMICs. However, most of the existing training components are often developed without input from prospective trainees, not specifically focused on neuropsychiatric genomics, and convened over a restricted time period. Therefore, a tailored approach, wherein researchers from the targeted populations continuously participate in defining neuropsychiatric training needs, might add value to the established methods through ensuring ownership and effectiveness of research training programmes.

The Global Initiative for Neuropsychiatric Genetics Education in Research (GINGER) programme seeks to address these limitations. GINGER is a collaboration between the Stanley Center for Psychiatric Research at the Broad Institute of Massachusetts Institute of Technology and the Harvard T H Chan School of Public Health, and several academic institutions in sub-Saharan Africa (University of Addis Ababa, Ethiopia; University of Cape Town and Stellenbosch University, South Africa; Moi University and the Kenya Medical Research Institute, Kenya; and Makerere University, Uganda). Comprising 17 investigators from these countries, GINGER aims to enhance the capacity of neuropsychiatric genetics research in Africa by training early-career researchers in genetics analysis, psychiatric phenotyping, epidemiology, bioinformatics, biostatistics, ethics, and manuscript and grant writing.

A unique feature of the 2 year GINGER programme is the active participation of the fellows and African mentors in selection of programme topics on the basis of the individual and collective needs of the fellows and their host institutions. Intra-African networking and collaborations are strongly encouraged to expand the training and capacity-building of the first GINGER cohort and to contribute to innovative research driven by African investigators. Furthermore, the initiative seeks to increase the awareness of key stakeholders and policy makers about the burden of neuropsychiatric disorders in sub-Saharan Africa.

The first cohort of GINGER fellows aims to establish an interdisciplinary group of African researchers, with the potential for sustainability beyond GINGER. Through continued conversations, skills building, and collaborative efforts between African institutions, this group will be equipped to produce first-class research equal to that produced in HICs. This Comment, therefore, serves as a call to action to develop and reaffirm the relationships needed to study neuropsychiatric genetics research in Africa. Improved characterisation of the prevalence and molecular causes of neuropsychiatric disorders in Africa might lead to development of population-specific and individual-specific treatment methods. These discoveries might ultimately improve global health-care strategies.
